# Out of (West) Africa—Who Lost in the End?

**DOI:** 10.4269/ajtmh.14-0753

**Published:** 2015-02-04

**Authors:** Piero Olliaro, Estrella Lasry, Amanda Tiffany

**Affiliations:** UNICEF/UNDP/World Bank/WHO Special Programme for Research and Training in Tropical Diseases (TDR), Geneva, Switzerland; Centre for Tropical Medicine, Nuffield Department of Medicine, University of Oxford, Churchill Hospital, Oxford, United Kingdom; Médecins Sans Frontières (MSF)/Doctors Without Borders, New York, New York; Epicentre and Médecins Sans Frontières (MSF) Switzerland, Geneva, Switzerland

On October 29, 2014, 4 days before the annual meeting of the American Society of Tropical Medicine and Hygiene (ASTMH) to be held in New Orleans, LA, meeting registrants received an e-mail letter from the Louisiana Department of Health and Hospitals stating “we have requested that any individuals that will be traveling to Louisiana following a trip to the West African countries of Guinea, Liberia, and Sierra Leone or have had contact with an Ebola-infected individual remain in a self-quarantine for the 21 days following their relevant travel history…we see no use in you traveling to New Orleans to simply be confined to your room.” This communication made it clear that those recently in countries experiencing the 2014 Ebola epidemic would not be able to participate in the meeting. The ASTMH sent their own communication stating that the Society did not agree with the State's policy, but had no choice but to abide. However inconvenient and upsetting this decision might have been, what really matters transcends the mere disturbance of long-planned schedules. More broadly, we lost on five levels.

First, there was a loss for evidence-based public health. Applying a blanket ban on travel for anyone within 3 weeks of arriving from Guinea, Liberia, or Sierra Leone, independent of the category of risk, was certainly expedient, but profoundly unjust, and also an inefficient public health measure. Those of us working in research and public health are tasked with producing evidence to be passed onto policy makers to help them make informed decisions. In these decisions they will have to weigh the evidence against a range of practical considerations—no easy chore. In this case, the decision was made “out of an abundance of caution.” Are our fellow citizens reassured? Do they feel better cared for? The majority most probably do, but this response appears proportional to the fear instilled by these very decisions—a dangerous vicious circle. Alarmism and misinformation call for drastic measures, and the process feeds itself. Enacting measures such as quarantine or a travel ban on everyone, when evidence says otherwise, sends contradictory messages to an already scared population. Disparate policies in different states and countries increase confusion.

Second, there was a loss considering the human factor. Think about physically and emotionally worn-out volunteers returning from a demanding experience in harsh conditions—where *en-passant* their own lives are at stake, and where they see so many people die. Imposing disproportionate restrictions (all the way to quarantine) will most certainly not help. These volunteers should feel welcome and cared for rather than ostracized. Beyond financial means, human resources are absolutely critical to help contain the Ebola epidemic and to provide care for the ill. Many would volunteer twice or more to serve in endemic countries, but will not feel encouraged if they have to go through 3-week restrictions each time they return home. Some would opt for hibernating in less hostile countries, but many lives will be disrupted. A related concern is how long these measures will be kept in place, and whether they will be updated as risks decrease.

Third, there was a practical loss, as we increased isolation of the affected countries, when what they actually needed was being supported and being heard. There is much that Ebola-afflicted countries can contribute. There are local doctors and nurses who have seen more Ebola patients than anyone else in the world. There are social workers who have been in direct touch with communities. There are those who have been applying control measures and have learned what works and what does not work. There are the potential trialists for the studies to come. And there are those who constitute the backbone of response and research. All of these residents of afflicted countries have been *de-facto* excluded from the international debate, where the decisions that affect their countries are made.

Fourth, there was a loss for all disease control in Ebola-afflicted countries. A blanket travel ban excluded all local experts, regardless of their area of expertise, from attending the ASTMH meeting. These countries desperately need a coordinated response to help them deal with all aspects of health, as Ebola has been the final blow for already shaky health systems. Yet, with the ban on attendance at the ASTMH meeting, experts on human immunodeficiency virus (HIV) infection, tuberculosis, malaria, and many other locally endemic diseases were unable to learn from their international counterparts.

Finally, there was a loss for international science. Scientific debate is stifled if those who have the “fingers on the pulse” are excluded. A gathering such as that provided by the ASTMH meeting is ideal for the exchange of experiences and information, and for discussions and debate to establish a research agenda toward finding solutions. We need all those with first-hand experience to contribute, but the travel ban prevented this.

Of course, meetings are not the full solution to international health problems, but they offer an important step in that direction. A silver lining, perhaps, is that increased light has been shone on the importance of evidence-based public health policy, with rational decisions replacing those based on fear.

